# Utilizing Machine Learning on Internet Search Activity to Support the Diagnostic Process and Relapse Detection in Young Individuals With Early Psychosis: Feasibility Study

**DOI:** 10.2196/19348

**Published:** 2020-09-01

**Authors:** Michael Leo Birnbaum, Prathamesh "Param" Kulkarni, Anna Van Meter, Victor Chen, Asra F Rizvi, Elizabeth Arenare, Munmun De Choudhury, John M Kane

**Affiliations:** 1 The Zucker Hillside Hospital Northwell Health Glen Oaks, NY United States; 2 The Feinstein Institutes for Medical Research Northwell Health Manhasset, NY United States; 3 Hofstra Northwell School of Medicine Hempstead, NY United States; 4 Cornell Tech Cornell University New York City, NY United States; 5 Georgia Institute of Technology Atlanta, GA United States

**Keywords:** schizophrenia spectrum disorders, internet search activity, Google, diagnostic prediction, relapse prediction, machine learning, digital data, digital phenotyping, digital biomarkers

## Abstract

**Background:**

Psychiatry is nearly entirely reliant on patient self-reporting, and there are few objective and reliable tests or sources of collateral information available to help diagnostic and assessment procedures. Technology offers opportunities to collect objective digital data to complement patient experience and facilitate more informed treatment decisions.

**Objective:**

We aimed to develop computational algorithms based on internet search activity designed to support diagnostic procedures and relapse identification in individuals with schizophrenia spectrum disorders.

**Methods:**

We extracted 32,733 time-stamped search queries across 42 participants with schizophrenia spectrum disorders and 74 healthy volunteers between the ages of 15 and 35 (mean 24.4 years, 44.0% male), and built machine-learning diagnostic and relapse classifiers utilizing the timing, frequency, and content of online search activity.

**Results:**

Classifiers predicted a diagnosis of schizophrenia spectrum disorders with an area under the curve value of 0.74 and predicted a psychotic relapse in individuals with schizophrenia spectrum disorders with an area under the curve of 0.71. Compared with healthy participants, those with schizophrenia spectrum disorders made fewer searches and their searches consisted of fewer words. Prior to a relapse hospitalization, participants with schizophrenia spectrum disorders were more likely to use words related to hearing, perception, and anger, and were less likely to use words related to health.

**Conclusions:**

Online search activity holds promise for gathering objective and easily accessed indicators of psychiatric symptoms. Utilizing search activity as collateral behavioral health information would represent a major advancement in efforts to capitalize on objective digital data to improve mental health monitoring.

## Introduction

Schizophrenia can be associated with significant impairment [1]. Although early intervention services have demonstrated the potential to improve outcomes [2], several challenges persist, limiting the established benefits of effective care. These include lengthy delays to early and accurate diagnostic ascertainment [3,4], as well as high rates of relapse, particularly during the early course of illness [5]. Under-recognized or misdiagnosed symptoms contribute to poorer outcomes such as social isolation, unemployment, and comorbid depression, anxiety, and substance abuse [6]. Furthermore, each new relapse can be associated with costly emergency room visits, psychiatric hospitalizations, family burden, medical complications, and suicide [7].

These challenges are compounded by the fact that psychiatry is still nearly entirely reliant on patient self-report. In contrast to all other areas of medicine, there are no reliable tests, biomarkers, or objective sources of collateral information available to inform diagnostic procedures or to assess mental health status [8-10]. Clinicians must therefore rely on subjective information, collected through patient and family interviews, to support diagnoses and make treatment recommendations. Technology offers the opportunity to collect objective digital data to complement self-reports and facilitate more informed treatment decisions [11-13]. Online search activity is a source of objective data with great potential.

Google search is one of the most popular websites worldwide, managing over 3 billion searches daily across over 600 million daily visitors [14]. Moreover, searching online has become a primary resource for youth seeking mental health–related information [15-20]. This is particularly true for stigmatized illnesses such as schizophrenia as the internet provides an easy and anonymous setting to gather information about symptoms and treatment options [21]. Importantly, online search engines store search activity as time-stamped digital records, offering a reliable source of objective, easily accessed, and detailed collateral information about an individual over an extended period of time.

Prior work has highlighted opportunities to utilize large-scale anonymized search logs to detect signals associated with the emergence and progression of medical illnesses [22]. For example, search activity, including content and patterns of use, has been used to identify individuals with lung cancer, Parkinson disease, and pancreatic cancer with high degrees of accuracy up to a year in advance of the diagnosis [23-25]. The success of these algorithms may lead to the development of a new generation of digital tools designed to assist in the screening and early identification of individuals at risk for medical conditions. Similar methods have been employed successfully in psychiatry using digital data extracted from social media sites [26-33]. However, few studies to date have explored the use of computational approaches to detect search patterns associated with psychiatric disorders [34]. Furthermore, while promising, online activity research thus far has been limited by the fact that it has been conducted primarily utilizing data extracted from anonymous individuals online who self-disclose having a particular diagnosis [35], and has yet to be carried out in real-world clinical settings using participant-contributed search data with clinically validated diagnoses.

Toward the goal of improving early diagnostic accuracy and relapse detection, we sought to conduct one of the first ecologically valid investigations into the relationship between online search activity and behavioral health. Specifically, we aimed to develop computational algorithms designed to accurately identify individuals with schizophrenia spectrum disorders (SSD) and to predict psychotic relapse based on internet search activity. We hypothesized that significant differences in the timing, content, and pattern of online search activity would differentiate participants with SSD from healthy volunteers, and that changes in these features would accurately predict a psychotic relapse in individuals with SSD.

## Methods

### Participants and Data Collection

Participants between the ages of 15 and 35 years were recruited from Northwell Health’s inpatient and outpatient psychiatry departments. Individuals with SSD were recruited primarily from the Early Treatment Program, Northwell Health’s specialized early psychosis intervention clinic (N=37). Additional participants diagnosed with SSD (N=7) were recruited from a collaborating institution located in East Lansing, Michigan. Recruitment occurred between March 2016 and December 2018. The study was approved by the Institutional Review Board (IRB) of Northwell Health (the coordinating institution) as well as by the local IRB at the participating site. Written informed consent was obtained for adult participants and legal guardians of participants under 18 years of age. Assent was obtained for participating minors. Healthy volunteers were approached and recruited from an existing database of eligible individuals who had already been screened for prior research projects at Zucker Hillside Hospital and had agreed to be recontacted for additional research opportunities (N=58). Additional healthy volunteers (N=21) were recruited from a southeastern university via an online student community research recruitment site. Healthy status was determined either by the Structured Clinical Interview for DSM Disorders [36] conducted within the past 2 years or the Psychiatric Diagnostic Screening Questionnaire [37]. If clinically significant psychiatric symptoms were identified during the screening process, participants were excluded.

Participants requested their search archive (known as “takeout”) through a simple process supported by Google. Participation involved a single visit during which all historical search activity was downloaded and collected. Each archive included a time-stamped record of search terms and browser history. Using hospitalization dates pulled from participants’ medical records, each participant's search data was segmented into 4-week periods immediately before and after each hospitalization. A 4-week period was selected as it represents an interval of time long enough to identify symptomatic changes [38,39] and also to contain sufficient online data required to train an algorithm [33,40]. For healthy participants (who did not have a hospitalization date), we randomly selected 4 weeks’ worth of search data to serve as a control.

### Diagnostic Classifier

A diagnostic classifier was built utilizing 4 weeks’ worth of search data immediately preceding the first psychiatric hospitalization. Data prior to the first hospitalization were selected to reduce the potential confounding influence of receiving a psychiatric diagnosis, being hospitalized, and receiving psychiatric interventions (such as therapy or prescriptions for psychiatric medications) on search activity. Concurrently, we built the diagnostic classifier using data closest to the time when the diagnosis is typically made (at the point of initial hospitalization) [41] to enhance the classifier’s potential clinical utility as a diagnostic support tool. A 4-week period before hospitalization was selected as it represents a period of time when psychotic symptoms would likely be most prominent. To match the data extraction period for both groups, we randomly selected 4 weeks’ worth of search data from each healthy participant to serve as a comparison group. This strategy also reduced possible effects of seasonality on search behavior. Participants diagnosed with SSD who did not have any search data in the 4-week period before their first hospitalization were excluded from this classifier. For healthy volunteers, if no search data existed in the randomly selected 4-week period, that participant was excluded.

### Relapse Classifier

A relapse classifier was built by segmenting the search data into 4-week periods of “relative health” and “relative illness.” Periods of relative illness were defined as the 4 weeks immediately preceding each relapse hospitalization, as it represents a period of time prior to hospitalization during which psychiatric symptoms are typically the most prominent. When less than 1 month existed between two consecutive hospitalizations, these data were not included in the classification model. Healthy periods were defined as the 4-week period immediately following discharge from a relapse hospitalization, as this represents a period of time when symptoms are typically better managed and less pronounced. If less than 2 months’ worth of search data existed between consecutive hospitalizations, these data were not included in the classifier, as we did not expect this period to represent a true period of relative health. Search data prior to the first hospitalization were not included in the relapse classifier. In total, 38 participants were included in the relapse classifier consisting of 51 periods of relative health and 42 periods of relative illness.

### Defining Features

We defined features of search content and search behavior using linguistic and temporal parameters. For linguistic features, we used linguistic inquiry and word count (LIWC) [42]. LIWC is a language analytic tool designed to capture and count the frequency of 51 different word categories, with established psychometric properties, including emotions, mood, cognition, thinking styles, and social concerns. A rich body of literature has identified associations between the use of LIWC categories and psychological health and illness [42,43]. We concatenated the Google search streams for the selected periods before passing them to LIWC as the input text for computing features. For the search behavioral features, we constructed histograms of length and frequency of queries using 1-hour bins as well as 4-day bins. This was done to explore search features that might accompany changes in circadian patterns associated with SSD. The 1-hour bin histogram helped to model finer changes in the length and frequency of search behaviors throughout the day, whereas the 4-day bin histogram was used to model broader changes in search behaviors. The 1-hour bin histograms were computed by creating 24 bins corresponding to each hour of the day and aggregating (through summation) each participant’s data across the 28 days. We chose hourly bins as this approach has been successfully implemented in prior research [27,44-46] exploring fluctuations in mood.

In addition, we included the total number of queries and the average query length for the 4-week period. We also included the standard deviation of the 4-day bin histograms (length and frequency) to represent the variation in search behaviors. Finally, we included directional changes in search behavior by computing first- and second-order statistics on the derivative of the 4-day histograms. All LIWC features were normalized based on the number of words in all searches concatenated for each participant, whereas the other features were normalized by subtracting the mean and dividing by the standard deviation. This process controls for any discrepancies in the feature values (ie, differences in the number of searches). A summary of all feature types along with the dimension of each feature is shown in Table 1.

**Table 1:** Feature categories along with the dimensionality of each feature type.

Feature type	Dimensions
24-hour histogram of length of queries with 1-h bin	24
24-hour histogram of frequency of queries with 1-h bin	24
32-day histogram of length of queries with 4-day bin	8
32-day histogram of frequency of queries with 4-day bin	8
SD of 4-day frequency of queries bins	1
SD of 4-day length of queries bins	1
Average of the derivative of 4-day frequency of queries bins	1
Average of the derivative of 4-day length of queries bins	1
SD of the derivative of 4-day frequency of queries bins	1
SD of the derivative of 4 day length of queries bins	1
Linguistic inquiry and word count	51
Total number of queries in 1 month	1
Average query length in 1 month	1

### Classifier Analyses

For both the diagnostic classifier and relapse prediction, we tested three classifiers: random forest (RF) [47], support vector machine (SVM) [48], and gradient boosting (GB) [49]. We used the standard python-based scikit-learn [50] library for evaluating classification performance. We performed hyperparameter tuning using a held-out validation dataset, which resulted in selection of optimal hyperparameters for the classifiers. For example, for SVM, we selected the radial basis function kernel over the standard linear kernel. Each classifier was validated using a 5-fold crossvalidation technique to avoid overfitting. To prevent bias in selection of healthy volunteer data, we tried 10 different iterations of randomly selected 4-week periods and found that the results were consistent. We calculated the average F1 score, average accuracy, and average area under the receiver operating characteristic curve (AUC) across 5 folds for each classifier. Since both diagnostic and relapse classifiers were trained on unbalanced datasets, we chose to evaluate the classifiers based on the AUC since it is a parameter that is agnostic to class imbalance [51].

### Feature Importance

A total of 123 features were used for each classifier. We used the permutation feature importance [52] method to compute the rank-ordered feature importance for each classifier. Under this method, feature importance is defined by the difference in the model’s score when the feature is randomly shuffled. Feature importance is proportional to the drop in the model score when the feature is shuffled. We used the AUC value as the model score. The feature importance was calculated on the validation set in 5-fold crossvalidation and the average score was computed across the 5 folds. We used this method as it is model-agnostic and enabled comparison of three different classifier models in an unbiased manner.

## Results

A total of 123 search archives (44 individuals diagnosed with SSD and 79 healthy volunteers) were available for analysis, and 116 (42 individuals with SSD an 74 healthy volunteers) met the inclusion criteria. Of these, 38 participants with SSD were available for the relapse classifier. An overview of the final dataset is shown in Table 2.

**Figure 1:**
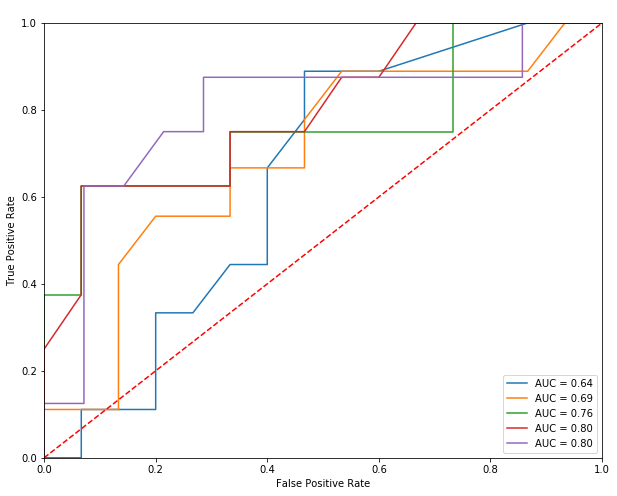
Receiver operating characteristic curves of the random forest diagnostic classifier for each of the 5 folds. AUC: area under the curve.

With respect to the diagnostic classifier (Table 3), the RF was selected for further feature analysis given its superior AUC compared to that of the other models. Figure 1 shows the receiver operating characteristic curves of the RF diagnostic classifier for each of the 5 folds. To explore consistency, this process was repeated 10 times with differing randomly selected 4-week periods of healthy volunteer data. Classifier performance remained consistent. Table 4 shows the quantity of search data provided per group for the diagnostic classifier.

**Table 2:** Participant demographics (N=116).

Characteristic		Value
Age (years), mean (SD)		24.38 (5.18)
**Sex, n (%)**		
	Male	51 (44.0)
	Female	65 (56.0)
**Race, n (%)**		
	Asian	18 (15.5)
	African American	32 (27.6)
	Caucasian	60 (51.7)
	Mixed/Other	6 (5.2)
Hispanic, n (%)		11 (9.5)
**Diagnosis, n (%)**		
	Schizophrenia	16 (13.7)
	Schizophreniform	13 (11.2)
	Schizoaffective	2 (1.8)
	Unspecified SSD^a^	11 (9.5)
Healthy volunteers, n (%)		74 (63.8)

^a^SSD: schizophrenia spectrum disorders.

**Table 3:** Diagnostic classifier results.

Classifier type	Mean F1	Precision (HV^a^)	Precision (SSD^b^)	Recall (HV)	Recall (SSD)	Mean Accuracy	Mean (SD) AUC^c^
Support vector machine	0.49	0.73	0.51	0.73	0.5	0.65	0.66 (0.09)
Random forest	0.54	0.75	0.72	0.86	0.48	0.73	0.74 (0.06)
Gradient boost	0.47	0.71	0.53	0.77	0.44	0.65	0.68 (0.09)

^a^HV: healthy volunteers.

^b^SSD: schizophrenia spectrum disorders.

^c^AUC: area under the receiver operating characteristic curve.

**Table 4:** Quantity of search data provided per group for the diagnostic classifier.

Metric	Healthy volunteers	Participants with SSD^a^
Total average queries (SD)	332.93 (298.1)	192.76 (214.19)
Weekly average queries (SD)	80.37 (71.92)	48.19 (52.91)

^a^SSD: schizophrenia spectrum disorders.

**Figure 2:**
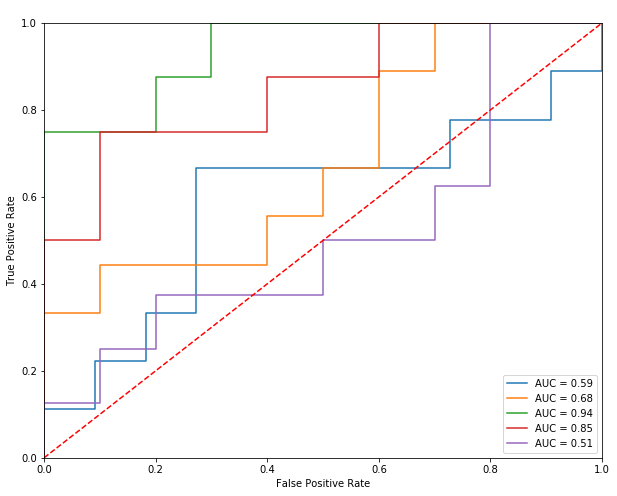
Receiver operating characteristic curves of the support vector machine relapse classifier for each of the 5 folds. AUC: area under the curve.

For the relapse classifier (Table 5), the SVM and GB models had the same AUC, and therefore both were considered for feature analysis. Further analysis of the feature importance of the SVM and GB relapse model revealed differing features. Herein, we report the SVM model as the identified features included search terms/themes that were deemed to be clinically interpretable and demonstrated some consistency with previous findings [33]; see Multimedia Appendix 1 for a comparison of the important features highlighted by both models. Figure 2 shows the receiver operating characteristics of the SVM relapse classifier for each of the 5 folds. The average F1 score for the SVM model was 0.36 and the average accuracy was 0.63. Table 6 shows the quantity of search data provided per group for the relapse classifier.

For each of the selected models, we calculated the top 20 features using the permutation feature selection method. The features sorted in decreasing order of feature importance for diagnostic and relapse classifies are shown in Table 7 and Table 8, respectively. For the two classifiers, both linguistic and behavioral features accounted for the top 20 features, indicating that both categories of features were important drivers of the classification result. Top features pertaining to the diagnostic classifier included a reduced search length between 12 am and 12 pm, lower overall number/frequency of search queries, as well as differences in the use of search terms/words from the “inhibition,” “positive affect,” and “anxiety” categories. Top features pertaining to the relapse classifier included differences in the use of search terms/words from the “sexual,” “health,” “hear,” “anger,” “sadness,” and “perception” LIWC categories, as well as reductions in search length and search frequency prior to a relapse hospitalization.

**Table 5:** Relapse classifier results.

Classifier type	Mean F1	Precision (HV^a^)	Precision (SSD^b^)	Recall (HV)	Recall (SSD)	Mean Accuracy	Mean (SD) AUC^c^
Support vector machine	0.36	0.61	0.77	0.92	0.26	0.63	0.71 (0.16)
Random forest	0.53	0.61	0.61	0.69	0.48	0.61	0.69 (0.09)
Gradient boost	0.57	0.66	0.63	0.75	0.53	0.65	0.71 (0.10)

^a^HV: healthy volunteers.

^b^SSD: schizophrenia spectrum disorders.

^c^AUC: area under the receiver operating characteristic curve.

**Table 6:** Quantity of search data provided per group for the relapse classifier.

Metric	Periods of relative health	Periods of relative illness
Total average queries (SD)	96.80 (98.77)	168.29 (250.18)
Weekly average queries (SD)	24.2 (11.17)	42.07 (39.9)

**Table 7:** Feature importance of diagnostic classifiers sorted by decreasing order of importance.

Diagnostic classifier features	Average feature importance (random forest)
Reduced search lengths between 8-9 am in participants with SSD^a^ compared to HV^b^	0.0315
Reduced search lengths between 6-7 am in participants with SSD compared to HV	0.0255
Length of queries from 23-20 days prior to first hospitalization is lower in participants with SSD compared to HV	0.0178
Reduced usage of “relative” LIWC^c^ features in participants with SSD compared to HV	0.0112
Variance in frequency of search lengths is lower in participants with SSD	0.0111
Reduced search lengths between 11am to 12 pm in participants with SSD compared to HV	0.0091
Reduced usage of “inhibition” LIWC features in participants with SSD compared to HV	0.0078
Reduced search lengths between 4 and 5 am in participants with SSD compared to HV	0.0073
Reduced usage of “quantifier” LIWC features in participants with SSD compared to HV	0.0072
Reduced search lengths between 1 and 2 am in participants with SSD compared to HV	0.0071
Reduced usage of “positive affect” LIWC features in participants with SSD compared to HV	0.0071
Reduced search lengths between 12 am and 1 am in participants with SSD compared to HV	0.0070
Reduced usage of “anxiety” LIWC features in participants with SSD compared to HV	0.0064
Lower overall number of queries in participants with SSD compared to HV	0.0062
Reduced usage of “preposition” LIWC features in participants with SSD compared to HV	0.0061
Reduced usage of “inclusive” LIWC features in participants with SSD compared to HV	0.0059
Frequency of search 19-16 days prior to first hospitalization is lower in participants with SSD compared to HV	0.0059
Reduced usage of “insight” LIWC features in participants with SSD compared to HV	0.0057
Number of queries between 2 and 3 am is lower in participants with SSD compared to HV	0.0056
Number of queries between 11 pm and 12 am is lower in participants with SSD compared to HV	0.0051

^a^SSD: schizophrenia spectrum disorders.

^b^HV: healthy volunteers.

^c^LIWC: linguistic inquiry and word count.

**Table 8:** Feature importance of relapse classifiers sorted by decreasing order of importance.

Relapse classifier features	Average feature importance (support vector machine)
Reduced length of queries during relapse periods	0.0688
Increased usage of “sexual” LIWC^a^ features during relapse periods	0.0523
Reduced length of queries 3-0 days prior to relapse hospitalization	0.0506
Reduced frequency of search activity during relapse periods	0.0263
Reduced usage of “health” LIWC features during relapse periods	0.0245
Increased usage of “hear” LIWC features during relapse periods	0.0224
Increased usage of “bio” LIWC features during relapse periods	0.0223
Increased searches in the 4 days before relapse hospitalization	0.0209
Reduced length of queries in the 7-4 days prior to relapse hospitalization	0.0196
Reduced frequency of searches 23-20 days prior to relapse hospitalization	0.0194
Increased usage of “percept” LIWC features during relapse periods	0.0186
Increased length of queries in the 31-28 days prior to relapse hospitalization	0.0162
Increased usage of “inclusive” LIWC features during relapse periods	0.0143
Denser searches during relapse periods	0.0140
Increased usage of “anger” LIWC features during relapse periods	0.0131
Reduced frequency of searches 19-16 days prior to relapse hospitalizations	0.0125
Reduced length of queries 11-8 days prior to relapse hospitalization	0.0105
Reduced usage of “sadness” LIWC features during relapse periods	0.0105
Increased usage of “indefinite pronoun” LIWC features during relapse periods	0.0104
Reduced frequency of searches 15-12 days prior to relapse hospitalization	0.0097

^a^LIWC: linguistic inquiry and word count.

## Discussion

### Principal Findings

We aimed to explore the feasibility of using collateral online search activity to support the diagnostic process and relapse detection in individuals with SSD. Our results indicate that important differences exist in the timing, frequency, and content of search activity in individuals with SSD compared to healthy volunteers. Furthermore, linguistic and behavioral shifts were identified in the month preceding a relapse hospitalization in individuals with SSD. This study demonstrates the promise of online search activity to potentially serve as collateral information informing diagnostic procedures as well as relapse identification strategies. Much like physicians routinely use medical imaging and blood tests to obtain objective and reliable clinically meaningful patient data, our results support the prospect of incorporating real-time machine learning–based extraction and analysis of online activity into psychiatric assessment.

### Features Relevant to the Diagnostic Classifier

Combining linguistic and behavioral features, the RF classifier distinguished individuals with SSD from healthy volunteers with an AUC of 0.74, suggesting that the integration of Google data with clinical information at the time of first hospitalization could potentially serve to improve the accuracy and reliability of clinical diagnoses [52]. Compared to healthy participants, those with SSD made fewer searches and their searches consisted of fewer words. Reduced search activity may represent declining interests and engagement with the environment [53-55]; as positive and negative symptoms of schizophrenia escalate, individuals with SSD may become less invested in their environment and increasingly internally preoccupied. Alternatively, reduced search activity could be related to cognitive deficits that are commonly associated with schizophrenia [56]. Given that cognitive changes may be subtle early in the course of illness [57], having an objective way by which to identify cognitive markers of SSD could contribute valuable information to the diagnostic process and inform treatment recommendations. Future research will need to explore precisely when changes first manifest online as well as their clinical significance. Online search data typically exist from the origin of an individual’s Google account, and the present results suggest that search data could prove to be particularly useful in charting the trajectory of an individual’s illness, as well as in contributing useful information about the timing of symptomatic changes.

Compared to healthy participants, those with SSD were significantly less likely to search for content related to “positive affect” (eg, “happy,” “good”), and less likely to search for content related to “anxiety” (eg, “nervous,” “tense”). These findings are consistent with the experience of low mood, apathy, and reduced emotional expression often associated with SSD [58,59]. These symptoms often predate the positive symptoms such as hallucinations and delusions, and therefore an objective method to identify them could help to overcome limitations of patient self-report to inform early intervention. Participants with SSD were also less likely to search using words from the relative (motion, space, time), inhibition (block, constrain), and inclusive (with, include) categories, and were less likely to use quantifiers (few, many, much) and prepositions (on, to, from). Determining the clinical significance of these differences requires additional research; however, they appear to be related to the complexity of the search query. Individuals with SSD often experience concrete thinking [60] in addition to the cognitive limitations noted above, and may therefore use less complex language when searching for information online.

### Features Relevant to the Relapse Classifier

Relapse periods could be distinguished from healthy periods with an AUC of 0.71. During a relapse period, participants with SSD were more likely to use words from the hear (heard, listen, sound), bio (eat, blood, pain), perception (see, touch, listen), and anger categories. They were less likely to use words related to health. These changes could be consistent with increasing delusions, hallucinations, and irritability during a psychotic relapse [61-63]. Previous work has identified changes in language use on social media that occur alongside escalating psychotic symptoms [33]. Thus, future research should aim to identify the point in illness progression at which linguistic shifts emerge online so as to make the best clinical use of this information.

Compared with periods of relative health, search length became shorter and the frequency of search activity decreased closer to the date of the relapse hospitalization. This could be indicative of a further decline in cognition function [56], or perhaps due to the presence of distracting internal stimuli. Fewer searches may also represent disengagement from one’s environment and reduced desire to ask questions and seek answers. This would be consistent with the avolition and negative symptoms commonly experienced by individuals with first-episode psychosis [59]. Additional research will be required to determine the precise clinical correlates.

### Limitations

The first limitation is that our approach was limited by our characterization of monthly periods of relative health and relative illness. The illness trajectory for individuals with SSD does not neatly fall into distinct segments of “health” and “illness,” and symptoms instead fluctuate over time. In addition, discharge from hospital does not necessarily mean full resolution of symptoms; therefore, we might have underestimated the potential differences between periods of illness and health. Furthermore, the inpatient hospitalization dates were obtained via medical records, and it is possible that some hospitalizations were missing from the record and therefore not included in our analyses. Related is the fact that the specific symptoms that define an exacerbation for each individual with SSD are often unique, and the impact of symptom heterogeneity on searches should also be explored in future work. To address these limitations and to improve the ability to find associations between online activity and psychotic symptoms, future studies need to monitor participants prospectively and utilize symptom rating scales to more accurately assess symptom changes and severity as well as to determine the specificity and sensitivity of our findings in comparison to other diagnostic groups. Additionally, future research will need to consider the potential influence of various life events, including search patterns associated with work and school.

Second, some individuals with SSD are diagnosed well before or long after the first psychiatric hospitalization, and therefore the generalizability of our diagnostic classifier is currently unknown. Ongoing efforts focused on understanding search behavior throughout the entire course of illness development, progression, and care should explore potential differences in those who are diagnosed at various time points.

Third, some participants were more active online than others, providing varying degrees of extractable data. An important question for future research will be how much search data is necessary to make reliable clinical predictions.

Fourth, the archives used for our analyses were collected retrospectively. Although retrospective collection eliminates the possibility of altering behavior as a result of being monitored, to achieve the goal of making real-time predictions, identifying clinically meaningful patterns in search data prospectively will be necessary.

Fifth, the eligibility criterion for age was between 15 and 35 years to reflect the inclusion criteria of the Early Treatment Program; however, adolescents may engage with search engines in a distinct manner compared to young adults, and age will need to be considered in future initiatives.

Sixth, we used nonlinear kernels in our classification models to accommodate nonlinear feature dependencies in the models. Although this is recommended for improved classification performance, it can also limit the interpretability of the features based on linear permutation methods. The feature permutation does not test for nonlinear permutations in features.

Seventh, for the purpose of this feasibility study, we considered both classification accuracy as well as feature interpretability in selecting our models. Further research with additional data from more participants is required to test the scalability of the selected classifiers and features as well as their generalizability to other online search engines beyond Google.

Finally, Google takeout only extracts search data collected while an individual is signed in to their account. Some participants may have searched for information while signed out, and these data would not have been captured in their archives.

### Conclusion and Prospects

Although search data alone are not sufficient to make a diagnosis or to predict a relapse, the integration of these data with information collected through traditional clinical means could be useful. Previous work has demonstrated that many people search for information online long before seeking help in person [9-11], and this study highlights the existence of a diagnostic signal in daily search patterns. Online services could one day facilitate the transition from information-seeking to help-seeking, hasten the diagnostic process, and help to reduce the burden of untreated psychosis. This approach could also be beneficial for relapse identification, enabling earlier intervention. Prior initiatives have explored the utility of smartphone sensor data (ie, geolocation, physical activity, phone usage, and speech), wearables, and social media activity to predict symptom fluctuations [44,64-66]. Our results demonstrate that user-generated search activity represents another potentially critical source of digital data contributing to the diagnostic process and relapse identification. Future work combining digital data from multiple sources will likely result in the most effective clinical tools. However, how to effectively and ethically incorporate personalized patterns of activity into clinical workflow are critical questions of inquiry. Interdisciplinary teams of researchers, clinicians, and patients must continue to work together on exploring challenges in ethics, privacy, consent, clinical responsibility, and data ownership. As our analyses become increasingly sophisticated and our ability to predict health information improves, stakeholders must develop standards to protect the confidentiality and the rights of this sensitive population, and ensure that the enabled technologies are used in the service of positive outcomes for the patients.

## Appendix

Multimedia Appendix 1 Top 20 features for the support vector machine (SVM) and gradient boost (GB) relapse classifiers in order of importance.

## Abbreviations

AUC: area under the receiver operating characteristic curve.
GB: gradient boost
IRB: Institutional Review Board
LIWC: linguistic inquiry and word count
RF: random forest
SSD: schizophrenia spectrum disorders
SVM: support vector machine
