# Supplementary material for: Utilizing Machine Learning on Internet Search Activity to Support the Diagnostic Process and Relapse Detection in Young Individuals With Early Psychosis: Feasibility Study
Source: JMIR Ment Health. 2020 Sep 1;7(9):e19348. doi: 10.2196/19348 (PMC7492982; doi:10.2196/19348)
Supplement: Multimedia Appendix 1 [file mental_v7i9e19348_app1.docx]

Supplementary Table 1. Top 20 features for SVM and GB relapse classifiers in order or importance.

| **Top 20 SVM classifier features** | **Top 20 GB classifier features** |
| --- | --- |
| Reduced length of queries during relapse periods | Reduced length of queries during relapse periods |
| Increased usage of "sexual" LIWC features during relapse periods | Shorter length of queries during relapse periods |
| Reduced length of queries 3-0 days prior to relapse hospitalization | Greater number of queries between 11 PM and midnight during relapse periods |
| Reduced frequency of search activity during relapse periods | Greater number of queries between 9 PM and 10 PM during relapse periods |
| Reduced usage of "health" LIWC features during relapse periods | Greater length of queries from 1 AM to 2 AM during relapse periods |
| Increased usage of "hear" LIWC features during relapse periods | Reduced length of queries from 9 PM to 10 PM during relapse periods |
| Increased usage of "bio" LIWC features during relapse periods | Reduced length of queries from 11 PM to midnight during relapse periods |
| Increased searches in the 4 days before relapse hospitalization | Increased length of queries from 6 AM to 7 AM during relapse periods |
| Reduced length of queries in 7-4 days prior to relapse hospitalization | Greater variance of the change in length of queries over a month in relapse periods |
| Reduced frequency of searches 23-20 days prior to relapse hospitalization | Reduced number of queries from 3 AM to 4 AM during relapse periods |
| Increased usage of "percept" LIWC features during relapse periods | Greater length of queries from 6 PM to 7 PM during relapse periods |
| Increased length of queries in 31-28 days prior to relapse hospitalization | Greater number of queries from 11 AM to noon during relapse periods |
| Increased usage of "inclusive" LIWC features during relapse periods | Greater length of queries from 8 PM to 9 PM during relapse periods |
| Denser search queries during relapse periods | Increased usage of "indefinite pronoun" LIWC during relapse periods |
| Increased usage of "anger" LIWC features during relapse periods | Greater variance of the length of search queries throughout the month during relapse periods |
| Reduced frequency of searches 19-16 days prior to relapse hospitalizations | Reduced number of queries in 3-0 days prior to relapse hospitalizations |
| Reduced length of queries 11-8 days prior to relapse hospitalization | Reduced number of queries closer to the relapse hospitalization |
| Reduced usage of "sadness" LIWC features during relapse periods | Greater length of queries from 11 AM to noon during relapse periods |
| Increased usage of "indefinite pronoun" LIWC features during relapse periods | Greater number of queries from 2 AM to 3 AM during relapse periods |
| Reduced frequency of searches 15-12 days prior to relapse hospitalization | Reduced length of queries in 11-8 days prior to relapse hospitalization |
